# Supplementary material for: Effect of Olive Pomace Oil on Cardiovascular Health and Associated Pathologies
Source: Nutrients. 2022 Sep 22;14(19):3927. doi: 10.3390/nu14193927 (PMC9573716; doi:10.3390/nu14193927)
Supplement: Supplementary file 1 [file nutrients-14-03927-s001.zip › nutrients-1882138-supplementary.pdf]

SUPPLEMENTARY TABLES

Table S1. Effect of olive pomace oil (OPO) and sunflower oil (SO) consumption on blood pressure.

	**Normocholesterolemic** *n= 31*		**Hypercholesterolemic** *n= 37*		p value		
	**OPO**	**SO**	**OPO**	**SO**	***Oil***	***N/H***	***N/H* Oil***
Systolic BP (mmHg)							
Initial	110 ± 2	111 ± 2	119 ± 3	119 ± 2	0.948	0.001	0.729
Final	110 ± 2	110 ± 2	119 ± 2	118 ± 2	0.984	0.000	0.811
Rate of change	0.00 ± 0.01	0.00 ± 0.01	0.00 ± 0.01	0 .00 ± 0.01	0.919	0.903	0.980
Diastolic BP (mmHg)							
Initial	73 ± 2	74 ± 2	80 ± 1	79 ± 2	0.853	0.000	0.821
Final	71 ± 2	73 ± 2	79 ± 1	78 ± 1	0.628	0.000	0.288
Rate of change	-3.00 ± 0.01	0.00 ± 0.01	0.00 ± 0.01	-1.00 ± 0.01	0.165	0.360	0.136

Values represent mean ± SEM. The table shows the initial (pre-treatment) and final (post-treatment) mean values. The rate of change was calculated from initial and final values as [(final value-initial value)/initial value] and expressed as percentage. Data were analyzed using a linear mixed model. p values in the first column correspond to the effect of taking the oil (olive pomace oil -OPO- or sunflower oil -SO-), those of the penultimate column to the effect of the group (normocholesterolemic or hypercholesterolemic), and the last column to the interaction of oil and group. Significance level was set at $p<0.05$. BP: Blood pressure.

Table S2. Effect of olive pomace oil (OPO) and sunflower oil (SO) consumption on antioxidant capacity and lipid peroxidation.

	**Normocholesterolemic** *n*= 31		**Hypercholesterolemic** *n*= 37		*p value*		
	**OPO**	**SO**	**OPO**	**SO**	***Oil***	***N/H***	***N/H* Oil***
FRAP (μM TE)							
Initial	499 ± 22	495 ± 22	612 ± 26	606 ± 29	0.547	0.000	0.580
Final	505 ± 22	507 ± 25	594 ± 29	587 ± 26	0.867	0.003	0.613
Rate of change	1.86 ± 0.02	2.73 ± 0.03	-3.31 ± 0.02	-2.56 ± 0.02	0.747	0.013	0.998
ABTS (μM TE)							
Initial	3057 ± 292	3055 ± 913	3757 ± 869	2948 ± 781	0.634	0.734	0.539
Final	3204 ± 897	3033 ± 879	3231 ± 782	3123 ± 756	0.867	0.944	0.964
Rate of change	17.52 ± 0.12	7.60 ± 0.06	- 2.63 ± 0.06	24.44 ± 0.14	0.295	0.793	0.141
ORAC (mM TE)							
Initial	20 ± 1	22 ± 1	19.7 ± 0.9	21 ± 1	0.397	0.628	0.864
Final	19 ± 1	20.5 ± 0.9	22 ± 1	20 ± 1	0.825	0.310	0.266
Rate of change	-1.68 ± 0.04	-0.82 ± 0.04	9.81 ± 0.04	1.42 ± 0.04	0.286	0.064	0.214
LDLox (ng/mL)							
Initial	122 ± 10	133 ± 10	177 ± 15	175 ± 14	0.762	0.000	0.558
Final	123 ± 10	129 ± 13	159 ± 16	177 ± 15	0.285	0.002	0.640
Rate of change	7.56 ± 0.07	1.49 ± 0.08	-9.96 ± 0.06	2.53 ± 0.05	0.318	0.252	0.173
MDA (nmol/mL)							
Initial	1.46 ± 0.07	1.41 ± 0.07	1.45 ± 0.07	1.40 ± 0.05	0.591	0.645	0.727
Final	1.37 ± 0.05	1.39 ± 0.06	1.43 ± 0.10	1.36 ± 0.04	0.685	0.702	0.406
Rate of change	-3.62 ± 0.03	0.66 ± 0.03	1.06 ± 0.03	-1.35 ± 0.03	0.936	0.507	0.278

Values represent mean ± SEM. The table shows the initial (pre-treatment) and final (post-treatment) mean values. The rate of change was calculated from initial and final values as [(final value-initial value)/initial value] and expressed as percentage. Data were analyzed using a linear mixed model. *p* values in the first column correspond to the effect of taking the oil (olive pomace oil -OPO- or sunflower oil -SO-), those of the penultimate column correspond to the effect of the group (normocholesterolemic or hypercholesterolemic), and the last column to the interaction of oil and group. Significance level was set at $p<0.05$. FRAP: Ferric ion reducing Antioxidant Power. ABTS: 2,2'-azino-bis (3-ethylbenzothiazoline-6-sulfonic acid). ORAC: Oxygen Radical Absorbance Capacity. LDLox: Oxidised low-density lipoprotein. MDA: Malondialdehyde.
